# Blood pressure shifts resulting from a concealed arteriovenous fistula associated with an iliac aneurysm: a case report

**DOI:** 10.1186/s40981-016-0057-2

**Published:** 2016-10-21

**Authors:** Shintaro Doi, Yoshiaki Motoyama, Hiromi Ito

**Affiliations:** 1 Department of Anesthesiology, Sanai Hospital, 4-35-17 Tajima, Sakura-ku, Saitama City, Saitama 338-0837 Japan; 2 Department of Anesthesiology, Tobu Chiiki Hospital, Tokyo, Japan

**Keywords:** Abdominal aortic aneurysm, Arteriovenous fistula, Hemodynamics, Iliac artery

## Abstract

**Background:**

A solitary iliac aneurysm (SIA) is more uncommon than an abdominal aortic aneurysm. The aneurysm is located in the deep pelvis and is diagnosed when it reaches a large size with symptoms of compression around adjacent structures and organs or when it ruptures. A definite diagnosis of an arteriovenous fistula (AVF) associated with a SIA is difficult preoperatively because there might not be enough symptoms and time for diagnosis. Here, we present a patient with asymptomatic rupture of SIA into the common iliac vein with characteristic blood pressure shifts.

**Case presentation:**

A 41-year-old man with a huge SIA underwent aortobifemoral graft replacement. Preoperatively, his blood pressure showed characteristic shifts for one or two heartbeats out of five beats, indicating that an AVF was present and that the shunt was about to having a high flow. During surgery, an AVF associated with the SIA was found to be concealed owing to compression from the huge iliac artery aneurysm, and the shunt showed a high flow, resulting in shock during the surgery. No complications were noted after aortobifemoral graft replacement. Postoperatively, we noted an enhanced paravertebral vein on computed tomography (CT), which indicated the presence of an AVF.

**Conclusions:**

Definite diagnosis of an AVF offers advantages in surgical and anesthetic management. We emphasize that a large SIA can push the iliac vein and occlude an AVF laceration, concealing the enhancement of the veins in the arterial phase on CT. Blood pressure shifts might predict the existence of a concealed AVF that has a large shunt. Even if the vena cava and the iliac veins are not enhanced on CT, anesthesiologists should carefully determine whether their distal branches are enhanced.

## Background

A solitary iliac aneurysm (SIA), an aneurysm that locates only in the iliac artery, occurs in 0.6 % of the case of an abdominal aortic aneurysm (AAA) [1], and many of the patients are free of symptoms. The aneurysm is located in the deep pelvis and is diagnosed when it reaches a large size with symptoms of compression around adjacent structures and organs or when it ruptures. An ilio-iliac arteriovenous fistula (AVF) occurs in less than 1 % of all cases of a common iliac artery aneurysm [2]. Because of its rarity and its various symptoms, the diagnosis and treatment of an AVF associated with a SIA are challenging to vascular surgeons and anesthesiologists. Here, we present a patient with asymptomatic rupture of a SIA into the common iliac vein with characteristic blood pressure shifts.

## Case presentation

**Fig. 1:**
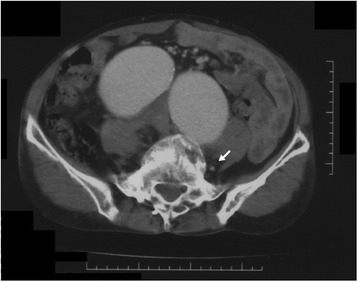
Computed tomography showing bilateral common iliac artery aneurysms and a dilated vena cava. Postoperatively, we could detect the enhanced paravertebral vein (*arrow*). The non-enhanced vena cava indicated an arteriovenous fistula located distal to the vena cava

**Fig. 2:**
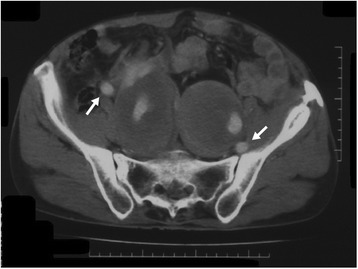
Aneurysms at the bilateral internal iliac arteries. The external iliac arteries (*arrows*) are intact. The iliac veins are indistinguishable from hematomas

A 41-year-old man (height, 178 cm; weight, 58 kg) with no medical history was admitted to the emergency unit on foot complaining of severe right inguinal pain. A right inguinal bulge was noted, and there was no lower limb edema. CT showed bilateral common iliac aneurysms (Fig. 1), and the internal iliac artery had a maximum diameter of 8 cm (Fig. 2). An AVF was not detected on CT, and chest radiography did not show heart or lung disorders. Emergent laparotomy was planned for the aneurysm rupture.

**Fig. 3:**
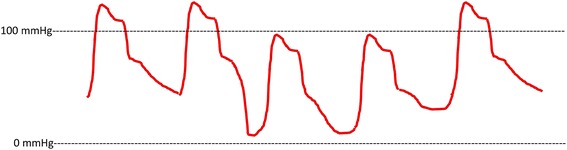
Schematic illustration of blood pressure shifts on arterial pressure wave. The arterial wave form deteriorates on diastolic runoff to achieve a far lower end-diastolic pressure. Following the falling, the next peak systolic pressure is measured lower than the latter. These shifts occur repeatedly, and the arterial wave form returns gradually to the former state

Preoperatively, his arterial pressure was stable with a systolic pressure of approximately 100 mmHg; however, there was a characteristic hemodynamic change (Fig. 3), with pressure shifting between 95/48 mmHg and 85/25 mmHg for one or two heartbeats out of five beats, with normal sinus rhythm.

**Fig. 4:**
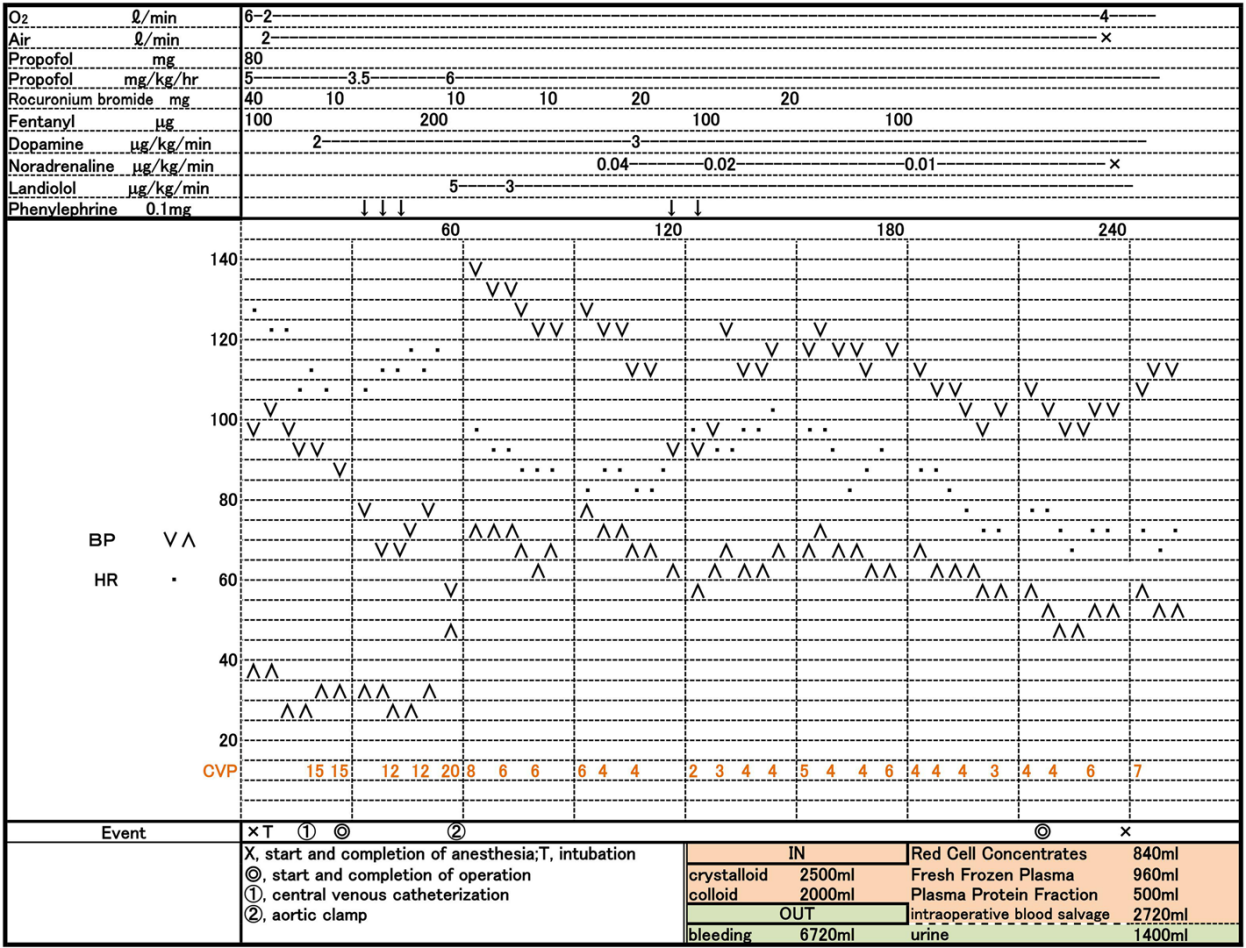
Overview of the anesthetic record

Anesthetic induction was successful. The detection of high central venous pressure (CVP) coincidently with reduction of arterial pressure indicated the presence of an AVF. During the surgery, his systemic hemodynamic condition worsened (Fig. 4). The CVP was initially 9 mmHg; however, it increased to 20 mmHg. His arterial pressure shifted frequently, and it reached a plateau of 60/50 mmHg. Urgent laparotomy and impetuous aortic clamping resulted in quick hemodynamic recovery, and the CVP reduced to approximately 6 mmHg. The right common iliac artery aneurysm showed a communication with the right common iliac vein. Therefore, he underwent aortobifemoral graft replacement, and no complications were noted. Postoperatively, he was not diagnosed with any connective tissue disorders, such as Ehlers-Danlos syndrome and Marfan’s syndrome.

### Discussion

We presented a case of acute aneurysmal rapture into the iliac vein. Sometimes, a definite diagnosis of an AVF associated with a SIA is difficult preoperatively because there might not be enough symptoms and time for diagnosis [3]. An AVF associated with an AAA has the following triad of symptoms: congestive heart failure, continuous abdominal bruit, and a pulsating abdominal mass [1]; however, these symptoms are noted in only 20–50 % of reported cases [4]. An AVF associated with a SIA might show lower limb edema as an additional feature; however, many cases do not have hemodynamic symptoms [4–6]. Definite diagnosis of an AVF offers advantages for surgical and anesthetic management.

Arterial pressure shifts rarely occur in a clinical situation. In the present case, these shifts indicated that an AVF was present and that the shunt was about to having a high flow. These hemodynamic changes could be explained by pooling of the transient increased shunt flow to a high-capacitance venous circuit and a decreased in preload, which can produce low arterial pressure at the next heartbeat. Simultaneously, an increase in venous return raised the blood pressure following a downward shift in the blood pressure. We hypothesized that the mechanical compression of a huge aneurysm should occlude the AVF and the fistula would appear by changing of lower limb posture, high blood pressure, or pulse of the aneurysm itself. The shift disappeared during the operative preparation, indicating that shunt dilation due to anesthetic agents and muscle relaxants decreased peripheral resistance, including aneurysmal compression at the vein.

**Fig. 5:**
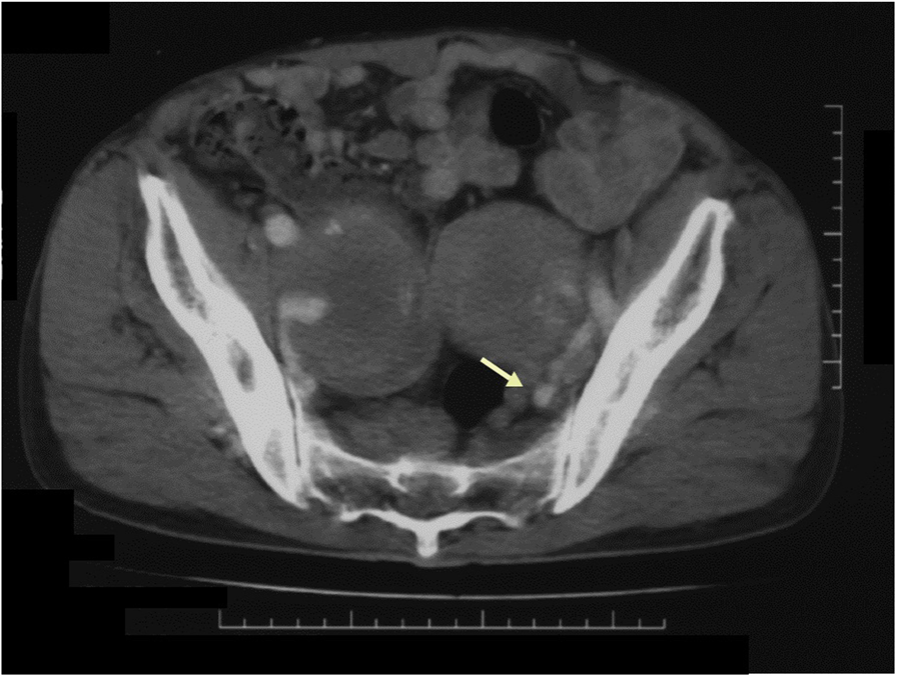
The left ascending lumbar vein (*arrow*) is enhanced

CT has been recommended to determine the subtypes, sizes, and complications of aneurysms [1, 3–6]. CT can contribute to the detection of an asymptomatic AVF associated with a SIA [4–6]. Although our case had hemodynamic catastrophe, the CT findings in our case were much fewer than the findings presented in previous reports. We were unsure of the presence of an AVF preoperatively because the dilated vena cava, which was not enhanced in the arterial phase, appeared to be apart from the aneurysm and the iliac veins were indistinguishable from the hematomas on CT. An asymptomatic aortocaval fistula due to abdominal aneurysmal compression [4] and an ilio-iliac AVF associated with a huge common iliac aneurysm [6] have been reported. Therefore, a huge iliac aneurysm could push the iliac vein aside and occlude the shunt easily. Postoperatively, we detected the enhanced left paravertebral vein (Fig. 1) and the left ascending lumbar vein (Fig. 5) on arterial-phase CT, and these were the only indications of the presence of an AVF on CT. The enhancement was noted at only the left paravertebral vein, although the laceration of the AVF was located at the right common iliac vein. The huge aneurysm obstructed the left common iliac vein and resulted in arterial inflow to the contralateral side. This supported the hypothesis that the aneurysm decreased shunt flow by compressing the adjacent vein.

## Conclusions

We report a patient with asymptomatic rupture of a SIA into the common iliac vein with characteristic blood pressure shifts. It was believed that the AVF did not cause a shock preoperatively because the laceration was small, the aneurysm ruptured into a relatively minor vein, and the huge aneurysm decreased shunt flow owing to compression of the common iliac vein. The blood pressure shifts indicated that the shunt of the AVF was about to open completely. Even if the vena cava and the iliac veins are not enhanced on CT, anesthesiologists should carefully determine whether the paravertebral vein and the ascending lumbar vein are enhanced. Detection of minor enhancement of these veins will help in the early diagnosis of a concealed AVF, thus preventing morbidity and mortality.

## Abbreviations

AAA: Abdominal aortic aneurysm
AVF: Arteriovenous fistula
CT: Computed tomography
CVP: Central venous pressure
SIA: Solitary iliac aneurysm
